# Implementation of a New Surgical Technique in a Gynecologic Oncology Centre: Sentinel Lymph Node Biopsy and Its Learning Curve in Endometrial Cancer

**DOI:** 10.3390/cancers17233813

**Published:** 2025-11-28

**Authors:** Michaela Koblížková, Petra Bretová, Luboš Minář, Michal Felsinger, Branislav Babjak, Libor Viktora, Petra Ovesná, Jitka Hausnerová, Eva Jandáková, Tatiana Stupková, Vít Weinberger

**Affiliations:** 1 Department of Obstetrics and Gynecology, University Hospital Brno and Faculty of Medicine, Masaryk University, 625 00 Brno, Czech Republic; koblizkova.michaela@fnbrno.cz (M.K.);; 2 Institute of Biostatistics and Analysis, Faculty of Medicine, Masaryk University, 625 00 Brno, Czech Republic; 3 Department of Pathology, University Hospital Brno and Faculty of Medicine, Masaryk University, 625 00 Brno, Czech Republic

**Keywords:** learning curve, endometrial cancer, sentinel lymph node, biopsy, staging

## Abstract

Objectives: This study aimed to evaluate the introduction of sentinel lymph node biopsy (SLNB) in early-stage endometrial cancer (EC), its learning curve, and factors influencing discrepancies between surgeons and pathologists. Methods: A single-centre retrospective observational study was conducted from June 2019 to December 2024 at the Department of Obstetrics and Gynecology, University Hospital Brno and Faculty of Medicine, Masaryk University. Patients with EC with complete preoperative staging and planned for extrafascial hysterectomy with bilateral salpingo-oophorectomy and SLNB were included. Bilateral detection rates were compared among two main surgeons—one senior fellow (A) and one junior fellow (B)—and other supervised fellows. Learning curves were assessed using detection rates and cumulative sum analysis. Risk factors for failed detection were analysed. Results: In 337 patients, overall bilateral detection rates ranged from 80–92%. Surgeon A achieved 80% success by the 30th procedure and 89% at the 74th. Surgeon B, trained under A’s supervision, reached 89% but later showed a decline after operating independently. The highest concordance with pathologists was noted for Surgeon A (94.6%), followed by B (92.2%) and others (84.9%). Discrepancies were mainly associated with the presence of fibroids (*p* = 0.005) and adenomyosis (*p* = 0.018). Conclusions: SNB in EC demonstrates an optimal learning curve that can be shortened through expert guidance. Extending supervised training to 30–35 procedures reduces post-independence decline and sustains high detection rates. Bilateral success, reflecting surgeon–pathologist concordance, is a strong indicator of the quality of a Gynecologic Oncology centre.

## Simple Summary

Sentinel lymph node biopsy (SLNB) enables precise evaluation of lymphatic spread in endometrial cancer, reducing the requirement for extensive surgical procedures. This study examined how surgeons at University Hospital Brno acquired the skills to perform this technique and identified the factors that influenced their success. Between 2019 and 2024, 337 women with endometrial cancer underwent hysterectomy with SLNB. The rate of successful lymph node detection increased with surgical experience and supervision. Surgeons achieved stable and high detection rates after about 30 procedures. Lower detection was observed in patients with fibroids or adenomyosis, conditions that can make mapping more difficult. The study shows that structured training and expert mentorship help surgeons master SLNB more efficiently. Extending supervised training to 30–35 procedures maintains accuracy and supports consistent, high-quality care for women with endometrial cancer.

## 1. Introduction

In 2025, the ESGO/ESTRO/ESP (the European Society of Gynaecological Oncology, the European Society for Radiotherapy and Oncology, and the European Society of Pathology) updated their guidelines for the management of endometrial cancer (EC), introducing necessary refinements in surgical staging [1]. Sentinel lymph node biopsy (SLNB) is now recommended for all patients with presumed uterus-confined disease, irrespective of risk group. Ultrastaging of sentinel nodes is advised to increase diagnostic accuracy. Systematic lymphadenectomy is reserved for patients with high-intermediate or high-risk disease in whom SLN (sentinel lymph node) mapping fails and may be considered for intermediate-risk patients. These updates reflect a shift toward minimising morbidity while preserving staging accuracy, as SNB significantly reduces complications associated with full lymphadenectomy, such as delayed recovery, lymphocyst formation, lymphedema, and lymphatic ascites [1,2]. The advantage of removing just a small number of lymph nodes is that their more thorough histopathologic examination by ultrastaging can detect macrometastases, micrometastases, and isolated tumour cells [3].

Historically, the radiocolloid 99Technetium (99Tc) was used for SLN labelling, either alone or in combination with methylene blue (blue dye). Both methods were gradually abandoned and replaced by ICG (indocyanine green) injection [4].

Regarding tracer application sites, the most used method with the highest detection rate was intracervical injection [5]. ICG is usually applied intracervically at two different depths (5 and 20 mm) from the cervical surface. Application can be performed in several ways, typically at two or four points [2].

However, the SLN must be successfully detected and removed bilaterally to assess the status of the lymph nodes. A problem arises when the surgeon evaluates the SLN detection as successful, despite definitive histology confirming adipose-only tissue, without lymphatic nodes present either unilaterally or bilaterally (“empty node dissection”) [6]. In both scenarios, in the cases of high-intermediate and high-risk tumours, this is considered an inadequate staging procedure [1]. Proper mastery of the technique requires individual expertise, which a learning curve can express. The learning curve is a mathematical concept that graphically expresses an individual’s progress in learning a new skill over time. Typically, it starts slowly as the learner gains initial understanding; then performance rapidly increases as competence develops, and finally plateaus when mastery is approached [7]. Only a few studies have investigated the learning curve of successful SLN detection in gynaecological malignancies.

This study aimed to analyse the implementation process of a new surgical technique in the Department of Obstetrics and Gynecology, University Hospital Brno and Faculty of Medicine, Masaryk University. The method was initially introduced by a single surgeon who subsequently trained colleagues. We are now able to retrospectively identify and analyse errors, formulate recommendations, and define quality indicators for other Gynecologic Oncology Centres within the learning process. As the primary objective, we established learning curves for two surgeons and identified concordance with the pathologist as a target parameter for evaluating the learning process. The secondary objective was to identify risk factors associated with empty node dissection that led to discrepancies between surgeons and pathologists. Based on real-world clinical data, we demonstrate that centralisation and proper guidance of experienced senior surgeons in oncologic surgery are essential, as they minimise or eliminate individual errors and enable rapid, systematic training of new oncogynaecologic surgeons within an experienced team.

## 2. Materials and Methods

This single-centre retrospective observational study consecutively enrolled patients undergoing surgery for endometrial cancer (EC) between June 2019 and December 2024. The study was approved by the University Hospital Brno Ethics Committee (approval number 02-190122/EK). All patients signed informed consent for the storage and scientific use of clinical data and histology samples. Inclusion criteria were defined as follows: histologically confirmed endometrial cancer; completion of full preoperative staging, consisting of expert transvaginal ultrasonography and pelvic–abdominal–thoracic CT scan to establish clinical TNM appropriately; surgical treatment always included extrafascial hysterectomy with bilateral salpingo-oophorectomy and SLNB. Exclusion criteria were as follows: radiological suspicion of enlarged and infiltrated lymph nodes, presence or suspicion of distant metastases; patients unable to undergo primary surgical treatment due to severe internal comorbidities.

All clinical data were obtained from the hospital’s information system. Patient characteristics, including age, BMI, and menopausal status, were collected. Surgical history was reviewed, focusing on procedures performed on the uterus and cervix. Information on clinical staging, disease extent, and tumour characteristics—including histotype, grade, myometrial invasion, cervical stromal invasion, and disease stage according to the 2009 FIGO (International Federation of Gynaecology and Obstetrics) classification—was also gathered. Additionally, data on the surgical management of sentinel lymph nodes and the female reproductive organs, as well as intraoperative and postoperative complications, were recorded.

We systematically monitored the success rate of performing SLNB bilaterally by two surgeons who introduced the technique and achieved the highest number of surgeries. Subsequently, we verified the histological results of the specimens by pathologists specialised in oncogynaecological pathology. Surgeon A was a highly experienced senior fellow in oncogynaecologic, on the verge of passing the board certification. He had extensive experience in laparoscopic surgery and introduced the SLN detection method into clinical practice at our institution. Surgeon B was a fellow in gynaecologic oncology with two years of experience in this programme. The other surgeons (“O”—others) were fellows in gynaecologic oncology with different lengths of experience in specialised training. They all worked under the supervision of specialists and other surgeons, including also surgeons A and B as assistants. No one achieved the number of surgeries that would have allowed us to set up the learning curve for another particular surgeon.

In the operating room, the patient in general anaesthesia was placed into a lithotomy position, and 4 mL of ICG was applied by the surgeon immediately before surgery. The application was performed intracervically at 5 mm and 20 mm depths at the 3 and 9 o’clock positions. A fluorescence imaging camera (Novadaq Pinpoint) was used to visualise the SLNs in the small pelvis. Re-injection was applied in case of failed ICG migration using the same protocol as for the primary application.

All SLNs were fixed in 10% buffered formalin, sliced at 2 mm lamellas, embedded in paraffin, and further examined using an ultrastaging protocol. This protocol consists of 4 μm thick consecutive sections of hematoxylin–eosin and cytokeratins (AE1/AE3), followed by two additional sections stained with hematoxylin–eosin at regular 200 μm intervals. This sequence of sections continued until there was no lymph node tissue left. We classified lymph nodes as follows: negative, isolated tumour cells (≤0.2 mm or single cells/clusters of cells ≤200 cells in a single SLN cross-section), micrometastasis (0.2–2 mm), and macrometastasis (>2 mm).

Standard statistics such as absolute and relative frequency, median, and interquartile range (IQR) were used for summarisation. Two measures of success were defined:

(i) Concordance—agreement between the surgeon’s intraoperative evaluation of SLNB and the pathologist’s assessment, and (ii) success—bilateral SNB confirmed by both surgeon and pathologist, i.e., concordance in cases with a positive finding according to the pathologist.

To identify independent predictors of successful bilateral SLN detection, we performed univariable logistic regression analyses for all potential predictors. Variables with clinical relevance and/or statistical significance were subsequently included in a multivariable logistic regression model. These included surgeon experience (number of previous SLNB procedures), age, BMI, postmenopausal status, history of caesarean section, >50% myometrial invasion, cervical stromal invasion, presence of uterine fibroids, adenomyosis, surgical approach, and operative time. Results were reported as odds ratios (OR) with 95% confidence intervals (95% CI).

Performance over time was graphically represented using learning curves. Learning curves were plotted using two methods: (i) the bilateral SNB detection rate is defined as the cumulative proportion of surgeries with successful bilateral SNB (as assessed by both surgeon and pathologist), while (ii) CUSUM (cumulative sum) statistic quantifies accumulated deviations from the mean performance and provides a graphical representation of the trend for experience. It is the cumulative difference between the individual points in time and the average of all points. For each surgeon, the CUSUM statistic was calculated as: ${CUSUM}_{n}=\sum_{i=1}^{n}\left(X_{i}-\bar{X}\right)$, where $X_{i}=1$ for successful bilateral detection and $X_{i}=0$ for failure, and $\bar{X}$ is the mean success rate of the entire series [8].

Further, a simulation was performed to model the learning curve for successful SLN detection depending on the length of supervision. The assumptions were based on real data from surgeon B when assisted by surgeon A and after becoming independent. The probability of success under the supervision of an experienced surgeon was set to 90%. After starting to operate independently, the probability of success depended on the number of supervised procedures, with a linear trend assumed and an incremental increase of 10% for every 10 assisted procedures. The target long-term probability of success after the learning phase was set to 80%. Simulations were conducted for 10, 20, 30, 40, and 50 assisted operations before independence. For each setting, 2000 repetitions were generated (bootstrap method). Cumulative success rates were calculated for each repetition, and the mean curve was reported.

This was a retrospective study that included all consecutive SNB procedures performed by the participating surgeons; therefore, no a priori power analysis was applicable. Cases with missing values were retained in descriptive tables and explicitly reported as “Unknown”. For multivariable logistic regression, only complete cases were used.

Analysis was conducted in R software (v4.3.2). All tests were performed as two-tailed at the 0.05 significance level.

## 3. Results

A total of 404 patients diagnosed with uterine cancer were assessed between June 2019 and December 2024. Of these, 337 patients who met the inclusion criteria and were enrolled in the study underwent surgery, including SLNB. Sixty-seven patients were excluded due to inoperability associated with severe internal comorbidities or an advanced stage of disease. The comprehensive characteristics of all included patients are summarised in Table 1.

In total, 90% of hysterectomies were performed laparoscopically, and 10% were performed via laparotomy. The study cohort included carcinomas ranging from stage I to IIIC according to the FIGO 2009 classification. The overall rate of bilateral SLN detection for all surgeons ranged from 80 to 92%. Postoperatively, all patients were stratified into final risk groups according to disease recurrence, and a decision on adjuvant therapy was made.

Regarding complications, perioperatively, one patient (0.5%) suffered a blood loss of 1000 mL due to a large vascular bundle damage (external iliac vein). One patient (0.5%) had a procedure complicated by ureter injury, and another patient (0.5%) suffered right-sided obturator nerve injury. Postoperatively, three patients (1.1%) developed symptomatic lymphocysts.

Out of 337 patients who underwent hysterectomy with BSO and SLNB, 125 patients were operated on by one of two surgeons. This was a crucial methodological step to establish the individual learning curve of SLN mapping. The remaining 212 surgeries were performed by other surgeons dedicated to the gynaecologic oncology programme at our department. None of them reached the sufficient number of surgeries needed to establish the learning curve. Therefore, these cases were excluded from that particular analysis.

We define successful SLNB detection as bilateral identification by the surgeon with confirmation by the pathologist. Bilateral success, confirmed by both surgeon and pathologist, represents a key quality indicator of the gynaecologic oncology centre.

There were no instances in which the surgeon assessed the procedure as unsuccessful, while the sampling was in fact successful. In two cases, the surgeon reported unilateral detection, whereas the pathologist confirmed bilateral detection. In 17 procedures (5%), the surgeon considered the SLNB successful, but the pathological assessment was less favourable (either unsuccessful or only unilateral).

Surgeon A achieved the highest success at 89.2%; all the others in the cohort achieved 79.5%. Differences between surgeons were borderline non-significant (*p* = 0.066) (Table 2).

We identified the presence of a fibroid (OR 0.46, 95% CI 0.27–0.8, *p* = 0.005) or adenomyosis (OR 0.48, 95% CI 0.26–0.9, *p* = 0.018) as the only factors influencing success, regardless of the surgeon.

**Figure 1:**
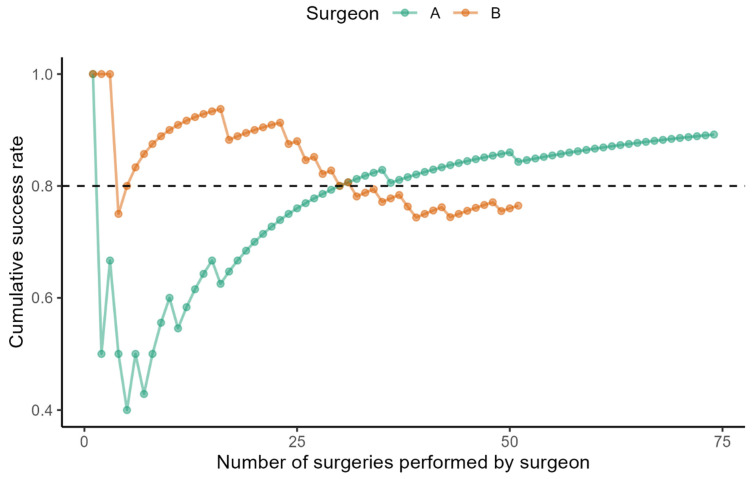
The learning curve of successful bilateral sentinel node biopsy.

The learning curve of the selected surgeons demonstrates how the cumulative success rate of bilateral SLNB evolved with increased experience (Figure 1).

Surgeon A achieved an 80% success rate at the 30th. He gained an 89% success rate after performing the 74th surgery (the last one evaluated). Surgeon B demonstrated a high success rate from the beginning, reaching 89%. Subsequently, his performance curve slightly declined. In the most recent observed operations (a total of 51 surgeries), the negative trend has been reversed, and his success rate has reached 75%.

**Figure 2:**
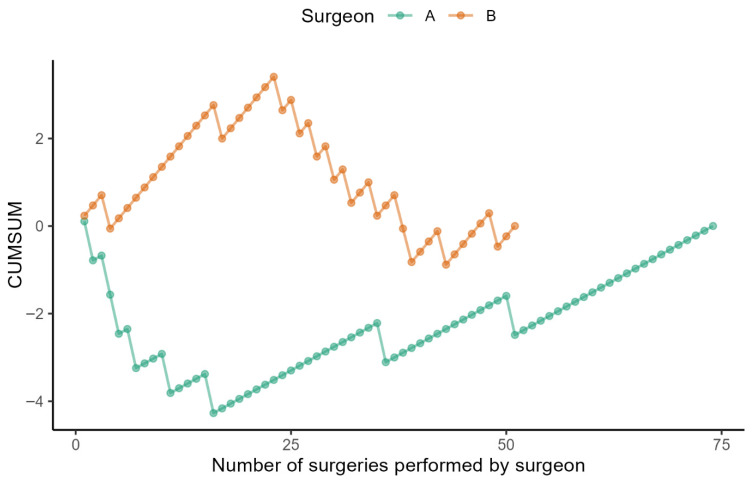
The learning curve of successful bilateral sentinel node biopsy according to CUSUM method.

When evaluating the cumulative difference between the observed number of successes and the average number of successes at each time to define the learning curve, we found a change from a decreasing trend to an increasing trend in Surgeon A on the 16th surgery (Figure 2).

**Figure 3:**
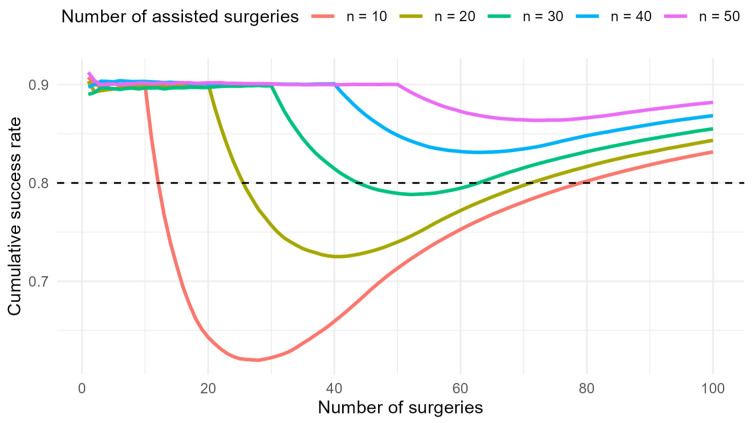
The simulated cumulative success rate for different lengths of assistance.

Surgeon B reached his peak performance after the 23rd procedure, after which a decline was observed. Improvement began following the 43rd procedure. This corresponds to the period when Surgeon B started performing procedures independently of Surgeon A. A positive trend became evident after approximately 20 operations. We attempted to estimate the optimal number of assistances provided by experienced Surgeon A to Surgeon B to prevent decline. We conducted a mathematical simulation to explore how longer assistance of Surgeon A to Surgeon B would influence the decline in performance of Surgeon B. The graph visualises the simulated cumulative success rate for different lengths of assistance. In practice, some decrease after independence is almost inevitable. If the assistance period had been longer (approximately 30–35 procedures), the decline would have been less marked, and the success rate would have been maintained above 80% (Figure 3).

## 4. Discussion

SLNB has become the standard surgical staging procedure for EC [1]. In addition, according to ESGO quality indicators for surgical treatment, SLNB is recommended in 90% of patients undergoing lymph node staging [9]. However, some institutions are just starting to implement this method to reach the recommended standards. Thus, we decided to share our experience with the learning process. So far, most of the publications have focused on the SLNB learning curve for CC (cervical cancer), and very few have targeted EC.

Regarding CC, Baeten et al. observed no learning curve in achieving bilateral SNB detection using robotic surgery, as they noted a stable bilateral detection rate of at least 80% when adhering to a standardised methodology. However, they used a combination of 99Tc and blue dye, which is considered outdated in EC nowadays [10].

Kim et al. presented a unicentric study with a limited number of only 80 SLNB using ICG in patients with CC (27) and EC (53). They assessed separately the left-sided (78%), right-sided (81%), and bilateral (66%) highest success rates achieved. When the cumulative success rate for both tumours was evaluated, there was a reversal to a positive success rate in the 27th procedure [11]. In our cohort, we evaluated only bilateral success, which achieved 79.5%.

According to another study, including seven surgeons, Tucker et al. demonstrated that every 10 additional procedures performed was associated with a 5% increase in the odds of successful SLN mapping, and every 11 additional procedures led to a 11% increase in the odds of successful SLNB. The plateau in rates of successful bilateral mapping was achieved after 40 cases [12]. For surgeon A in our study, every 10 additional procedures in the early learning phase (up to 30 operations) increased the likelihood of bilateral SLN retrieval by 15%. No standard learning curve was observed for the others, though such a pattern could be expected across all operators, under the supervision of experienced surgeons. These authors chose a different methodology to describe the learning curve. They analysed surgeries annually over the 7-year study period. While this study excludes the first three procedures of each surgeon from individual learning curves due to variability in cumulative estimates, no surgeries were excluded from our learning curve analysis.

In our study for EC, the reversal point in Surgeon A was at the 16th surgery, and he achieved an 80% success rate at the 30th surgery. However, Surgeon B reached peak performance after the 23rd procedure, followed by a decline once he began operating without supervision. It then took 20 procedures before the trend turned favourable. The question remains whether a more extended period of supervision by a more experienced surgeon would have mitigated the subsequent decline. According to our mathematical model, approximately 30–35 supervised procedures would have been required to maintain Surgeon B’s success rate of bilateral detection above 80%. These findings highlight the importance of adequate supervision in surgical training. Extending the period of assistance allows junior surgeons to acquire technical skills more effectively and shortens the critical period of independent practice during which patients are at higher risk of unsuccessful procedures.

In a recent publication, Gedgaudaite et al., eight differently experienced surgeons were compared, and the overall absolute success rate of SLNB was 89.5% [6]. According to the study results, at least 30 surgical procedures are required to achieve a 75% success rate. The surgeons reached a success rate from 57 to 88.9%, and their outcomes were not dependent on the length of experience. The success rates of our surgeons were 89% for surgeon A, 77% for surgeon B, and 77% for the remaining surgeons. Surgeon A achieved 70% and 80% success rates on the 20th and 30th surgeries, respectively, while surgeon B reached a 75% success rate on the 51st surgery. The more experienced surgeons progressively supported and assisted the remaining surgeons. Consequently, the remaining surgeons demonstrated high success rates from the outset. It is evident that in a training centre, where a sufficient number of experienced surgeons are available in assisting roles, the inexperience of the primary surgeon can be effectively mitigated. This finding strongly supports the rationale for centralisation and the establishment of centres of excellence in gynecologic oncology, as such procedures cannot be performed optimally in every setting.

Additionally, we focused on one of the most disturbing types of failure—a specimen identified and removed as an SLN by a surgeon remains without any lymph node on the final pathology report (“empty node dissection”). This could represent more than one-third of mapping failure cases [13]. The probable explanation for this phenomenon is that since ICG is an albumin-bound substance, it absorbs more interstitial fluid into the lymphatic channels by oncotic pressure; consequently, the lymphatics look swollen and bigger and can be incorrectly identified as a lymph node [14]. The presence of underlying lymphatic injury or surgery plays a more critical role in visible swelling patterns than ICG itself [15].

The main issue with not recognising failure during the surgery is not providing side-specific lymphadenectomy in high-intermediate and high-risk cases, which leads to inadequate staging. In our cohort, among 21 attempts (tentamen), bilateral lymphadenectomy should have been performed in 7 cases (5 classified as high-intermediate risk and 2 patients as high risk, based on definitive histology). In the group where only unilateral detection was achieved, unilateral lymphadenectomy should have been performed in 4 high-intermediate risk patients and 6 high-risk patients.

There are several options for decreasing the risk of “empty node dissection”. The surgeon should assess the shape of the coloured specimen-SLN tends to be rounder than swollen lymphatic vessels. Inspecting specimens with different modes of fluorescence camera (black and white mode or Colour Segmental Fluorescence mode) also helps distinguish between SLN and lymphatics. Another option is to gently palpate the specimen ex vivo, or to perform perioperative frozen section examination [13].

Regarding factors influencing bilateral SLNB success, in a recent meta-analysis, Raffone et al. retrospectively evaluated six studies (a total of 1345 patients) and the presence of adenomyosis came out as statistically insignificant [16]. In our study, besides the surgeon’s experience, we observed that adenomyosis or fibroids may negatively influence the concordance between surgeons and pathologists.

The risk factors for SLNB failure proven in the recent meta-analysis are the application of ICG less than 3 mL and enlarged lymph nodes [17]. We avoided these factors by thorough preoperative staging and using an amount of ICG of 4 mL. Previous practical experience performing systematic lymphadenectomies is an indisputable advantage when providing SLN mapping. Due to the incorporation of SLNB into clinical practice, it is evident that the performance of systematic retroperitoneal lymphadenectomies is decreasing. The question is whether a lack of experience with retroperitoneal surgeries would affect the learning curve of next-generation surgeons.

The limitations of our study must be acknowledged. This is a retrospective, single-institution analysis, which inherently carries a risk of bias and limits the generalisability of the findings. Randomisation in a retrospective study was not possible. Ideally, the study should be methodologically conducted by comparing a team of expert laparoscopic oncogynaecological surgeons versus trainees and versus trainees plus an assisting expert. Then it would be possible to evaluate learning curves for the individual groups of doctors and determine the optimal combination of surgeons, setting a general process for learning this new technique for other centres. While this represents a methodological limitation, it may also be considered a strength, as the study reflects authentic clinical conditions in which a new technique is implemented. Surgeons must gradually acquire and refine the necessary skills. Importantly, we identified the presence of fibroids and adenomyosis as the only independent factors significantly associated with reduced mapping success, irrespective of surgeon experience.

Nonetheless, this study provides practical insights relevant for other centres initiating SLNB programmes. Notably, our data indicate that even when a junior surgeon appears to achieve rapid proficiency. Ongoing supervision by a more experienced surgeon remains essential to ensure optimal outcomes and should not be withdrawn prematurely.

Hospitals must take a more methodological approach to the teaching process, the introduction of new surgical techniques, and to publish these efforts, so that robust and generally applicable conclusions can be established.

## 5. Conclusions

SLN detection and biopsy are currently considered the preferred method in the surgical staging of newly diagnosed EC, with a clear emphasis on a minimally invasive surgical approach. Our findings highlight the importance of structured supervision by an experienced surgeon during the initial phase of learning new clinical skills. Based on our experience, we recommend performing 30–35 SLNB procedures under the guidance of a more experienced surgeon. Adequate supervision not only facilitates the acquisition of technical skills but also mitigates the risk of performance decline once independent practice begins. Successful bilateral SLN detection—defined as agreement between the surgeon and the pathologist in achieving bilateral sampling—represents a strong indicator of the quality of a gynaecologic oncology centre. Furthermore, to ensure the maintenance of surgical expertise and to uphold consistently high standards of patient care, centralisation of cases within specialised centres is essential.

## Figures and Tables

**Table 1:** Patients and tumour characteristics.

	Overall (*N* = 337)	A (*N* = 74)	B (*N* = 51)	O (*N* = 212)	*p*-Value ^1^
Age (years), median (IQR)	65 (58–73)	66 (61–73)	65 (57–73)	65 (58–72)	0.652
BMI, median (IQR)	32.0 (27.7–36.0)	33.7 (30.1–38.0)	32.0 (29.0–37.0)	31.0 (26.0–35.1)	0.003
Menopausal status					0.564
Premenopausal	29 (8.6%)	4 (5.4%)	5 (9.8%)	20 (9.5%)	
Postmenopausal	307 (91.4%)	70 (94.6%)	46 (90.2%)	191 (90.5%)	
Unknown	1	0	0	1	
Cone biopsy	17 (5.0%)	6 (8.1%)	3 (5.9%)	8 (3.8%)	0.292
Myomectomy	3 (0.9%)	1 (1.4%)	1 (2.0%)	1 (0.5%)	0.310
Caesarean section	32 (9.5%)	7 (9.5%)	2 (3.9%)	23 (10.8%)	0.323
Adnexal surgery	22 (6.5%)	3 (4.1%)	2 (3.9%)	17 (8.0%)	0.452
Previous Pelvic RT	0 (0.0%)	0 (0.0%)	0 (0.0%)	0 (0.0%)	>0.999
Previous CHT	4 (1.2%)	0 (0.0%)	1 (2.0%)	3 (1.4%)	0.466
Histotype					0.124
Endometrioid (incl. mucinous)	312 (92.6%)	68 (91.9%)	44 (86.3%)	200 (94.3%)	
Non-endometrioid	25 (7.4%)	6 (8.1%)	7 (13.7%)	12 (5.7%)	
Grade					0.697
Low grade (1 + 2)	282 (83.7%)	61 (82.4%)	41 (80.4%)	180 (84.9%)	
High grade	55 (16.3%)	13 (17.6%)	10 (19.6%)	32 (15.1%)	
Myometrium infiltration					0.997
<50%	250 (74.2%)	55 (74.3%)	38 (74.5%)	157 (74.1%)	
>50%	87 (25.8%)	19 (25.7%)	13 (25.5%)	55 (25.9%)	
Cervical infiltration	52 (15.4%)	9 (12.2%)	10 (19.6%)	33 (15.6%)	0.524
LVSI					0.299
No	214 (63.5%)	50 (67.6%)	36 (70.6%)	128 (60.4%)	
Yes focal	123 (36.5%)	24 (32.4%)	15 (29.4%)	84 (39.6%)	
Yes substantial	0 (0.0%)	0 (0.0%)	0 (0.0%)	0 (0.0%)	
MELF	49 (14.5%)	11 (14.9%)	8 (15.7%)	30 (14.2%)	0.958
Myoma	113 (33.5%)	27 (36.5%)	9 (17.6%)	77 (36.3%)	0.033
Adenomyosis	60 (17.8%)	13 (17.6%)	6 (11.8%)	41 (19.3%)	0.446
FIGO					0.800
IA	228 (67.9%)	49 (66.2%)	34 (68.0%)	145 (68.4%)	
IB	37 (11.0%)	7 (9.5%)	5 (10.0%)	25 (11.8%)	
II	28 (8.3%)	5 (6.8%)	5 (10.0%)	18 (8.5%)	
IIIA	12 (3.6%)	5 (6.8%)	1 (2.0%)	6 (2.8%)	
IIIB	4 (1.2%)	0 (0.0%)	0 (0.0%)	4 (1.9%)	
IIIC	27 (8.0%)	8 (10.8%)	5 (10.0%)	14 (6.6%)	
Unknown	1	0	1	0	
Prognostic risk group					0.655
Low	188 (56.1%)	38 (51.4%)	29 (56.9%)	121 (57.6%)	
Intermediate	46 (13.7%)	8 (10.8%)	8 (15.7%)	30 (14.3%)	
High-intermediate	43 (12.8%)	12 (16.2%)	4 (7.8%)	27 (12.9%)	
High	58 (17.3%)	16 (21.6%)	10 (19.6%)	32 (15.2%)	
Unknown	2	0	0	2	
Approach					0.645
LSK	303 (89.9%)	67 (90.5%)	44 (86.3%)	192 (90.6%)	
LPT	34 (10.1%)	7 (9.5%)	7 (13.7%)	20 (9.4%)	
Histol. SLN status					<0.001
Negative	266 (82.6%)	63 (88.7%)	40 (80.0%)	163 (81.1%)	
ITC	19 (5.9%)	0 (0.0%)	2 (4.0%)	17 (8.5%)	
Macrometastasis	13 (4.0%)	6 (8.5%)	5 (10.0%)	2 (1.0%)	
Not done	24 (7.5%)	2 (2.8%)	3 (6.0%)	19 (9.5%)	
Unknown	15	3	1	11	

^1^ Kruskal–Wallis rank sum test; Fisher’s exact test; Pearson’s Chi-squared test.

**Table 2:** Concordance between surgeons and pathologists.

	Overall (*N* = 337)	A (*N* = 74)	B (*N* = 51)	O (*N* = 212)	*p*-Value ^1^
SLNB detection according to surgeon					0.158
Tentamen	21 (6.2%)	2 (2.7%)	2 (3.9%)	17 (8.0%)	
Unilateral	32 (9.5%)	4 (5.4%)	8 (15.7%)	20 (9.4%)	
Bilateral	284 (84.3%)	68 (91.9%)	41 (80.4%)	175 (82.5%)	
SLNB detection according to pathologist					0.201
None	23 (6.8%)	2 (2.7%)	3 (5.9%)	18 (8.5%)	
Unilateral	44 (13.1%)	6 (8.1%)	9 (17.6%)	29 (13.7%)	
Bilateral	270 (80.1%)	66 (89.2%)	39 (76.5%)	165 (77.8%)	
Concordance					0.054
No	40 (11.9%)	4 (5.4%)	4 (7.8%)	32 (15.1%)	
Yes	297 (88.1%)	70 (94.6%)	47 (92.2%)	180 (84.9%)	
Positive bilateral SLNB success					0.066
No	69 (20.5%)	8 (10.8%)	12 (23.5%)	49 (23.1%)	
Yes	268 (79.5%)	66 (89.2%)	39 (76.5%)	163 (76.9%)	

^1^ Fisher’s exact test; Pearson’s Chi-squared test.

## Data Availability

The data that support the findings of this study are available from the corresponding author upon reasonable request.
